# Editorial for the Special Issue “Advances in Multi-Omics for Functional Genomics Studies and Molecular Breeding”

**DOI:** 10.3390/cimb48080763

**Published:** 2026-07-27

**Authors:** Chen Chen

**Affiliations:** Shaanxi Engineering Research Centre for Conservation and Utilization of Botanical Resources, Xi’an Botanical Garden of Shaanxi Province, Institute of Botany of Shaanxi Province, No. 17 Cuihua South Road, Xi’an 710061, China; chenchen@xab.ac.cn

## 1. Introduction

Plant functional genomics has entered an era of unprecedented resolution and scale, driven by the rapid evolution of sequencing technologies and the integration of multi-omics approaches. The convergence of genomics, transcriptomics, proteomics, metabolomics, and epigenomics has fundamentally transformed our ability to dissect the molecular architectures underlying plant growth, development, stress responses, and secondary metabolism [1,2]. For non-model plants, which constitute the vast majority of agricultural, horticultural, and forestry germplasm but lack the extensive genomic resources of Arabidopsis or rice, multi-omics tools are particularly transformative, enabling gene discovery and functional characterization at a pace previously unimaginable. Equally important, the knowledge generated through multi-omics platforms is increasingly being translated into molecular breeding strategies, from gene editing to marker-assisted selection, accelerating the development of improved crop varieties [3,4].

This Special Issue of *Current Issues in Molecular Biology*, entitled “Advances in Multi-Omics for Functional Genomics Studies and Molecular Breeding”, was conceived to capture the breadth and depth of research at this intersection. We received contributions from researchers across multiple countries, reflecting the global and collaborative nature of contemporary plant molecular biology. Following rigorous peer review, fourteen manuscripts were accepted for publication, comprising twelve original research articles, one review, and one short communication. The contributions span a diverse array of species, experimental systems, and methodological frameworks, collectively illustrating how multi-omics integration is advancing both fundamental understanding and applied outcomes in plant science.

## 2. Molecular Mechanisms of Plant Abiotic Stress Responses

Abiotic stress remains one of the most pressing challenges facing global agriculture under climate change; several contributions tackle this problem through multi-omics lenses [5]. Hong et al. cloned a heat shock protein gene *ClaHSP17.4* from watermelon and demonstrated through heterologous expression in *Arabidopsis* that it significantly improves thermotolerance by enhancing antioxidant capacity and osmotic regulation [6]. Zhou et al. integrated transcriptomic and metabolomic analyses of rapeseed under salt stress during germination, revealing that phenylpropanoid metabolism and hormone signal transduction represent early and dominant adaptive pathways [7]. Su et al. compared heat-tolerant and heat-sensitive sesame varieties at the transcriptomic level, pinpointing differential regulation of antioxidant defense systems, auxin homeostasis, and heat shock proteins as key determinants of stress resilience [8]. Fu et al. identified 25 NAC transcription factor family members in the halophyte *Suaeda glauca*, most of which were upregulated under salt stress, underscoring the value of mining extremophile plants for novel genetic resources [9]. Additionally, a study on apple rootstock *Malus halliana* elucidated the role of WRKY69 transcription factor in iron deficiency tolerance, demonstrating its positive regulatory function through enhanced antioxidant enzyme activity and iron reduction [10].

## 3. Genetic and Metabolic Bases of Organ Pigmentation and Quality Traits

Anthocyanin biosynthesis has long served as a paradigm for studying gene–metabolite relationships, and the contributions here extend this framework to new species and tissues. Zhong et al. employed metabolomics and transcriptomics to dissect pod color variation in purple-pod pea, identifying both structural genes in the flavonoid pathway and regulatory transcription factors orchestrate pigment deposition [11]. Li et al. leveraged transcriptomic profiling across pigmented and non-pigmented potato varieties to reveal co-expression networks linking specific MYB transcription factors to anthocyanin biosynthetic genes, offering molecular targets for color-directed breeding [12]. Beyond pigments, Yang et al. used multidimensional transcriptomics to clarify the respective contributions of malate metabolism and vacuolar acidification to the low-acid phenotype in apples, providing a theoretical basis for flavor improvement in pome fruits [13]. Ko et al. developed a novel fruit-color and leaf-shape mutant of *Citrus unshiu* through gamma irradiation, coupled genome resequencing with marker development, and established a specific selection marker for this unique variety [14].

## 4. Genome Sequencing, Evolution, and Mutagenesis

The assembly and annotation of organellar genomes remain foundational for phylogenetic reconstruction and cytoplasmic inheritance studies [15]. Zhang et al. reported the first complete mitochondrial genome of *Platycrater arguta*, a rare and endangered Tertiary relict shrub, providing essential data for conservation strategies and evolutionary studies [16]. Skuza et al. resolved the chloroplast genome sequence of *Secale strictum* subsp. *africanum*, a putative ancestor of cultivated rye, contributing to our understanding of domestication history and genetic diversity in Triticeae species [17]. On the mutagenesis front, Chen et al. mapped chemically induced pigmentation mutations in rice variety IR64, revealing that retrotransposon insertions in *OsC1* and *3GT* genes restore anthocyanin biosynthesis, illustrating the dynamic interplay between transposable elements and phenotypic variation [18].

## 5. Biotic Stress Resistance and Developmental Regulation

The de novo transcriptome assembly of cedar (*Cedrela odorata*) by Guzmán et al. under herbivore attack enabled the identification of differentially expressed genes associated with defense signaling, toxin production, and volatile compound biosynthesis, establishing a foundation for marker-assisted selection of pest-resistant genotypes [19]. In developmental biology, Song et al. provided a comprehensive review of photoperiodic gene networks regulating heading date in rice, consolidating decades of progress on how environmental cues integrate with endogenous genetic programs to control flowering time, and highlighting emerging roles of epigenetic modifications in this process [20].

Looking forward, several challenges and opportunities merit attention. First, while multi-omics integration has become technically feasible, the biological interpretation of large-scale datasets remains a bottleneck. Advances in machine learning and network biology will be essential to move beyond correlative associations toward predictive models of gene function and phenotype [21]. Second, the gap between gene discovery in non-model plants and their deployment in breeding programs needs to be narrowed. High-throughput transformation platforms, genome editing tools such as CRISPR-Cas9, and genomic selection strategies must be adapted to a broader range of species to realize the full potential of functional genomics [22,23,24]. Third, the environmental plasticity of gene expression and metabolic profiles necessitates more extensive multi-environmental studies to ensure that laboratory findings are robust under field conditions [25]. Finally, the ethical and regulatory frameworks surrounding transgenic and gene-edited crops continue to evolve, and the plant science community must engage proactively with these discussions to facilitate the responsible translation of research outcomes [26].

In summary, this Special Issue brings together a diverse set of studies that collectively demonstrate the power of multi-omics approaches in advancing plant functional genomics and molecular breeding. From stress tolerance mechanisms to quality trait formation, from organellar genome evolution to pest resistance, the contributions reflect the vibrancy and breadth of contemporary research in this domain. We hope that the findings and perspectives presented here will stimulate further inquiry and foster collaborations that bridge basic science and agricultural application.

We would like to extend our sincere gratitude to all authors for their outstanding contributions, to the expert reviewers for their thorough and constructive evaluations, and to the editorial team at *Current Issues in Molecular Biology* for their professional support throughout the publication process.

## Data Availability

No new data were created or analyzed in this study. Data sharing is not applicable to this article.
